# How the European Union responded to populism and its implications for public sector reforms

**DOI:** 10.1007/s43508-022-00034-1

**Published:** 2022-02-15

**Authors:** Edoardo Ongaro, Fabrizio Di Mascio, Alessandro Natalini

**Affiliations:** 1 Faculty of Business and Law, The Open University, Milton Keynes, UK; 2 Interuniversity Department of Regional and Urban Studies and Planning, University of Turin, Turin, Italy; 3 Department of Law, Economics, Politics and Modern Languages, Lumsa University of Rome, Rome, Italy

**Keywords:** Populism, European governance, European Union, Public sector reform, Italy

## Abstract

A major shift occurred in the European Union (EU) approach to tackle the apparently unstoppable rise of populist parties across European countries and to preserve the integrity of the EU polity. EU economic governance seems to have shifted from a logic of conditionality to a logic of solidarity underpinned by a pan-European strategic view allowing EU governance to support and enable public sector reforms at the national level. By investigating the case of Italy as an EU member state, we find that the European governance shift occurring in the wake of the COVID-19 pandemic was largely mediated by the mutating character of Italian populism. A logic of conditionality which was largely centered around EU governance was largely shifted to a logic of solidarity taking into account political conditions in the member countries.

## Introduction

A major shift occurred in the European Union (EU) approach to preserve the integrity of the European single market and the EU polity (Armingeon et al., forthcoming; Ferrera et al., 2021) in the face of the combined, and dramatic, challenges posed by the follow up of the economic and fiscal crises triggered by the financial crisis of 2008, the migration crisis (uncontrolled inflow of migrants) in the mid-2010s, and the rise of populist parties across Europe over the 2010s (in turn fuelled by the combined economic-fiscal and migration crises). This shift got underway in the second half of the 2010s and unfolded with dramatic rapidity in 2020 in the midst of the COVID-19 epidemic. To stem the increasing gaps between the economic performance of EU member states and to tackle the growing risks of ‘weaker’ countries being ‘left behind’ became EU policy priorities. Because economic discontent domestically and perceived abandonment by an allegedly indifferent and faceless EU had been deemed to be key drivers of the populist tide (Bauer et al., 2021), diminishing economic discontent within its Member States and reducing the perception of indifference by the EU was deemed essential by the EU’s establishment. In pursuing such objectives, the EU economic governance shifted from a logic of conditionality (whereby funding and other forms of EU support to weaker EU countries were tied to strict conditions being foisted on the recipient country) to a logic of solidarity (involving re-distribution of resources by leveraging collectively backed issuance of common debt, the so-called ‘Next Generation’ Recovery and Resilience Fund) underpinned by a pan-European strategic view.

EU governance influences not just national economies but also the dynamics of public sector reforms internal to member states. Ongaro and Kickert, (2020) minted the notion of ‘EU-driven public sector reforms’ to capture this phenomenon. There has been an observable shift in EU governance from being focused on constraining public spending by individual countries and shrinking the public sector to an EU governance that supports and enables public sector reforms at the national level through technical advice provided by the Structural Reform Support Service—SRSS in the period 2017–2020. This service made it possible to support public authorities in the Member States in their efforts to design reforms policies according to their own priorities and strengthen their capacity to develop and implement administrative reforms, as well as to benefit from good practices and examples of their EU peers. Funding could be applied for by committing to the implementation of structural reforms that had been identified in the context of the European Semester’s cycle and laying out a detailed set of reform targets and intermediate milestones.

This study aims at analysing the change in EU governance, understood as a response to populism, and its implications for public sector reform processes in the EU Member States. Our research questions can be formulated as follows:

–RQ1: what explains the shift in EU economic governance?
–RQ2: what are the implications of the shift in EU economic governance for public sector reforms in EU Member States?

The overall theoretical framework and narrative of the paper is summarised in Table 1 and outlined in the remainder of this section.

**Table 1:** Framework of the paper

RQ and main explanandum	Theoretical reference(s)	Overall narrative about the role of populism in governance shift and policy change
RQ1: what explains the shift in EU economic governance?	Adapted Two-Level Game theory (Putnam, 1988) (overarching reference theory) Political Leadership theory (Hartley, 2012) Window of opportunity for policy change (Kingdon’s model, 2004) Policy and organizational learning theories (Dunlop & Radaelli, 2018)	The shift in EU economic governance is explained as a response by EU actors to the effects of the economic and fiscal crisis, which fuelled the rise of populist parties
RQ2: what are the implications of the shift in EU economic governance for public sector reforms in EU Member States?	Contextual influences on public management reforms, policy implementation theories (Pollitt & Bouckaert, 2017; Ongaro, 2009; Ongaro & Valotti, 2008)	Populist parties, both in government and in opposition, with their mutating and opportunistic policy stance affect the dynamics of the process of public sector reform

We use a dynamic “Two-Level Game” (TLG} framework originated by Putnam, (1988) for international negotiations but adapted here to the study of reform processes involving EU and domestic levels of governance. In the original framework, developed in and for the field of International Relations, the main actors were domestic: the international level was seen as an arena but not a set of actors in their own right; however, the latter is indeed the case of the EU, which is why we employ an adapted version of TLG. Drawing on De La Porte & Natali, (2014), we put particular emphasis on how economic and institutional circumstances influence reform processes. While the TLG framework focuses on the interaction between the domestic and international levels of governance, we focus on those intervening factors that influence when and how EU actors and domestic actors interact in the process of public sector reform, our main explanandum.

The first intervening factor is the ‘vulnerability’ of the European Monetary Union (EMU—the international policy regime defining the governance of the common currency of most EU Member States: the Euro): the stability and even long-term existence of the Euro is dependent on the economic and budgetary status of its individual Member States, a level of interdependence not reflected in the original TLG framework. The second intervening factor are the dynamics of the interrelated EU and domestic politics, which comprise divergences of interests between EU institutions and Member States’ governments (second modification to TLG), as well as between political parties at the domestic level (this one being an original feature of TLG which we keep in our framework). These, in turn, affect EU adjustment pressures upstream and policy reform processes downstream. EU adjustment pressures include formal procedures, conditionality and “backroom diplomacy”. Formal procedures refer to policy coordination under the Stability and Growth Pact (SGP) and reinforced surveillance under the Excessive Deficit Procedure (EDP) in the case of a non-compliance with fiscal targets. Conditionality consists of financial help provided by the EU in exchange for structural reforms. Backroom diplomacy refers to informal negotiations used by the EU and the most powerful Member States to convince domestic policy-makers to follow instructions from the EU. In our framework, the EU adjustment pressures and intervening factors affect the decision to enact public sector reforms at the national level.

Empirically, we focus on the case of Italy, which is noteworthy in a number of respects. First, Italy is a large Member State of the EU, both in terms of its economy and of its representation within the EU institutions, meaning that EMU vulnerability is affected by the country’s political and budgetary conditions (Badell et al., 2019). Second, Italy is a country where domestic politics have traditionally led to policy inertia in the field of public sector reform. Consequently, EU adjustment pressures have been intense. Third, the ‘implementation gap’ of public sector reforms (Ongaro & Valotti, 2008) has grown further over the first two decades of the 2000s, characterized by the presence of populist parties in government (Verbeek & Zaslove, 2016). During this time, and unique in Europe, Italy witnessed three coalition governments dominated by populist parties, including the second government led by Prime Minister Giuseppe Conte, which is the one that managed the COVID-19 crisis throughout 2020, followed in February 2021 by the government chaired by Mario Draghi, former President of the European Central Bank, whose ‘grand coalition’ government also encompasses two supposedly former populist parties in its parliamentary majority.

The process of “mutating populism”, i.e., the interaction among diverse populist parties that emerged in the last 2 decades as incarnations of a consolidated anti-establishment ethos, is essential for understanding domestic politics in Italy. As populist parties entered coalition governments, new populist actors exploited the inefficacy of governments affected by the antagonism and paralysis resulting from the incoherence of policy positions deriving from the coalition partners diverse electoral interests (Di Mascio et al., 2021).

In the next two sections we describe and critically review the rise of populism in Europe and especially in Italy. In the following section we analyse policy and governance change at the EU level, interpreted as a response to a series of crises especially involving fiscal and immigration issues in which populism, from the perspective of the EU leadership, played a key role as part of the problem. The influence of the new European Union economic governance on the dynamics of public sector reforms is then analysed through the discussion of the case of Italy (thereby contributing to a consolidating stream of literature—Badell et al., 2019, Di Mascio et al., 2021; Kickert & Ongaro, 2019; Ongaro & Kickert, 2020—originally advocated by Ongaro, 2014).

Our findings highlight that the influence of the European governance shift occurred in the wake of the COVID-19 pandemic has been largely mediated by the mutating character of Italian populism. This characteristic of Italian populism appears to be a critical element in impeding the shift in EU governance to translate into reforms that potentially could enhance Italian state, administrative and policy capacity at the national level (Painter & Peters, 2005).

## The fiscal and migration crises and populism in Europe

The global ascendance of electoral populism—understood as the electoral success of populist political parties outside the mainstream of politics—has driven a burgeoning strand of scholarly work in recent years (Peters & Pierre, 2019). Yet, a commonly accepted definition of populism is still lacking, with disagreements regarding categorization and boundaries between its different manifestations (Caiani & Graziano, 2016). This is probably also due to the variety of forms of populism and their ideological vagaries (Ivaldi et al., 2017). In this discussion populism should be defined as a set of ideas that not only depicts society as divided between the “people” as a force for good and the custodian of national virtues versus a “corrupt elite” scheming to frustrate the people’s legitimate expectations to a better life but also claims that governing is about respecting a popular will that is allegedly manifest and non-contradictory in its expression and originates from the “real” people of the country (Mudde & Rovira Kaltwasser, 2018).

Following this approach, populism can be a set of doctrines that lacks a programmatic centre and “borrows” from extant ideologies, often mixing right—and left-wing elements. By combining populism with other ideologies, populists can politicize grievances that are relevant in a specific context. This explains why we observe the formation of very different types of populist forces across place and time, combining populist elements with a diverse range of “host ideologies” such as nationalism, neoliberalism, and socialism. It also implies that, in reference to the European party systems, populism can be an attribute of parties on the culturally conservative right as well as the anti-capitalist left. Right-wing exclusionary versions of populism usually rely on nativism to depict a narrowly conceived ethnically-based understanding of who “the real people” are, whereas left-wing inclusionary types of populism normally rely on socialism to advance a definition of “the real people” that embraces the socioeconomic underdog (Mudde & Rovira Kaltwasser, 2013).

Despite their ideological differences, populist parties of the left and right have in common a diffidence, if not outright hostility, to the process of European integration (van Kessel, 2016). This is connected to the technocratic and elitist nature of EU-decision making, which all populists are prone to oppose (Taggart, 2004). Opposition to European integration can be broken down into “hard” and “soft” alternatives. The hard variant refers to the outright rejection of the idea of European integration and opposition to EU membership. The soft variant supports the general idea of European integration, but it is pessimistic about the current and future practice of the integration process. Additional arguments against ‘Europe’ tend to vary between different types of populist parties. The radical right typically portrays the EU as a project that threatens the sovereignty of the nation and, through the opening of borders, cultural homogeneity whereas radical left parties typically describe European integration as a neoliberal project that encourages a “race to the bottom” in terms of welfare entitlements and working conditions (Rooduijn & van Kessel, 2019).

The European economic and sovereign debt crisis between 2009 and 2014 has been defined as a “transformational moment in the history of the EU” with unprecedented consequences for the politicization of European integration (Otjes & Van der Veer, 2016). Populist parties across Europe typically exploited the crisis as an opportunity to reiterate their objections to European integration as the external constraints on national governments’ room for manoeuvre on welfare spending became more obvious (Pirro et al., 2018). Populist political forces are a threat to the EU as a project of transnational cooperation and governance. Opposition at the national level towards European integration reached an extent unmatched in its previous half a century of existence, leading to new political dynamics at both the EU and national level. (Notable has also been a trend toward politicizing other international organisations such as the World Trade Organization or the International Criminal Court, (de Vries et al., 2021). The political effects of the economic crisis stretched beyond the short-term electoral punishment of incumbent parties resulting from the economic hardship affecting domestic populations (Hernández & Kriesi, 2016). Populist parties benefited from the exodus of voters away from mainstream parties and this process accelerated the destabilization of the party systems across Europe (Hobolt & Tilley, 2016). The Eurosceptic position adopted by most populist parties put pressure on national governments and made it more difficult to reach agreement on policy issues. The electoral success of populist forces also increased parliamentary fragmentation, and this made the formation and maintenance of stable coalitions increasingly difficult.

The economic crisis, however, had an asymmetric impact on party systems across Europe depending on whether the countries affected by the crisis were subject to EU fiscal management conditions. Research assessing the impact of externally imposed austerity in southern European ‘debtor’ countries has shown there to be negative effects on both satisfaction with democracy and electoral support for mainstream, particularly left-leaning, parties (Ruiz-Rufino & Alonso, 2017; Alonso & Ruiz-Rufino, 2020). This means that the Euro-crisis boosted the level of politicization of the issue of European integration: in other words, notably in southern European countries where citizens observed bailouts and other informal forms of external intervention on their national economies by unelected EMU institutions, the political issue of whether EU integration is beneficial or not became salient and contested: a major change from previous decades of euro-enthusiasm for EU integration in these countries. Citizens perceiving an increasing loss of autonomy of national governments vis-a-vis EMU institutions opted for more radical left populist parties that vociferously opposed the external constraints imposed by EU membership on national economic policy. It is questionable, however, as to whether the increased Eurosceptic vote for the Southern European radical left populist parties is an expression of a backlash against Europe. It more plausibly could be interpreted as the expression of an alternative vision of a more solidary Europe, but one that clashes with the vision that prevails in some of the Northern European countries. In these northern ‘creditor’ countries voters backed radical right-wing populist parties campaigning on the repatriation of national powers and disapproval of economic solidarity.

The refugee crisis in 2015 enhanced the Euroscepticism—meaning a political stance unfavourable to European integration—both of the populist parties on the far right in Northern Europe, often opposition parties, and of the more established conservative centre-right parties in Eastern Europe, often parties holding executive office. In both of these regions, nationalism and anti-immigration sentiments gained traction (Kriesi, 2020). Yet, the issue of European integration is treated flexibly by radical right populist parties, which perform frequent and significant shifts, particularly when entering government, as this issue is far less salient among their supporters than immigration (McDonnell & Werner, 2019).

Overall, the issue of European integration has become much more politicized in the aftermath of the fiscal and the refugee crises. However, the positions that populist parties on the cultural right and the economic left have taken over the last decade in Europe are profoundly different in many respects (Hutter & Kriesi, 2019), notably on whether they adopt an inclusionary conception of the people or an exclusionary one. What is even more striking is how differently they have governed (Vachudova, 2021). As self-proclaimed political outsiders, populist parties in Europe are not naturally part of the governing elites. Nonetheless, it has become increasingly common for populist parties to participate in government. While squaring their anti-establishment brand with a role in office may be complicated for populist parties, some have displayed a marked acceptance of compromises. As shown in the next section, this is particularly true with regard to Italy, a country marked by the diversity of populist parties and by their repeated presence in government over the last 2 decades.

## Mutating populisms in Italy

Uniquely in Europe, Italy witnessed five coalition governments dominated by populist parties in the period 1994–2021. More specifically, Italy is a case of “mutating populism” where diverse populist parties emerged (Forza Italia, or “Go Italy”, FI; Lega Nord, or “Northern League”, LN; the Movimento Cinque Stelle, “Five Star Movement” FSM; Fratelli d’Italia, or “Brothers of Italy” FdI) as different incarnations of a consolidated anti-establishment ethos. The ability to enter into coalition agreements despite, or perhaps because of, their highly varied and fluid ideologies has been a key unifying feature of Italian populist parties. This has triggered a political environment in which populist actors react to their fellow populist parties. As these parties enter coalition governments, new populist actors exploit the inefficacy of governments torn by antagonism and paralysis resulting from the incoherence of the policy stances of the coalition partners (Verbeek & Zeslove, 2016). The populist parties outside of the government then turn to their advantage the poor performance of the extant government to harvest the electoral consensus previously held by the other populist parties.

The success of populist parties is in part traceable to certain long-term determinants of political dissatisfaction characterizing modern Italy (Ongaro et al., forthcoming). The poor delivery of public services resulting from traits typical of Southern European bureaucracies—politicization of administrative elites, formalism and legalism, clientelism and corruption, among others—is one of the key factors of chronic dissatisfaction with political parties and elites. Another key contextual feature is, however, imposed rather than institutional in nature. This is the austerity that has been imposed by the Eurozone governance on the Italian budget in the aftermath of the 2008 global financial meltdown (Badell et al., 2019; Di Mascio et al., 2017). The imposed austerity has been both a blessing and a curse for populist actors. On the one hand, austerity helped fuel dissatisfaction that stimulated support for populist parties while on the other hand it limited leeway to bring about major changes in economic policy when populist parties entered government. Austerity has also contributed to a major shift in the Italian party system after the outbreak of the crisis. Although Italy is one of the founding Member States of the EU and traditionally one of the most pro-European countries, it has had a polarised party system regarding the EU. Euroscepticism has increased and its political leaders have become more vocal in that regard (Conti et al., 2020).

However, not all Italian populist parties are Eurosceptical. Silvio Berlusconi’s political incarnations (Forza Italia—FI, 1994–2008, and again since 2013; The People of Freedom—PDL, 2008–2013) are examples of alleged mainstream populism that have not been always Eurosceptical. Berlusconi proved remarkably resilient over the years as the parties he led were the dominant force of the centre-right coalition that won the national elections in 1994, 2001 and 2008. FI is affiliated with the European People’s Party, a pro-European political transnational coalition that coalesces centre-right parties in the European Parliament. Over the years, FI moved back and forth from a broad but vague pro-Europeanism to a contingent Euroscepticism, rooted mainly in the defence of national sovereignty through intergovernmentalism. The sovereign debt crisis marked a turning point in terms of party attitude towards the EU as FI made use of such slogans as ‘less Europe in Italy’ and promoted stories of an EU conspiracy against Italian interests (Conti et al., 2020). Yet, this does not qualify FI as a challenger party, since it cooperated with the austerity measures implemented by the Monti technocratic government (2011–2013).

The main radical-right wing populist party, the Northern League (LN), which joined the centre-right coalition led by Berlusconi in 1994, 2001 and 2008, refused to support the Monti government and became the strongest parliamentary opponent of the new government. Ever since its establishment in the early 1990s the LN has mainly expressed a pessimistic view of the EU. It has progressively shifted from ethno-regionalism (initially advocating the secession of the northern regions from the rest of Italy) to nativism by including xenophobia and ‘law and order’ in its discourse. The election of Matteo Salvini to be the new party federal secretary in December 2013 catalysed the programmatic shift from regionalism to nationalism that was complemented by a very strong criticism of the EU (Pirro & van Kessel, 2018). However, the LN never appeared to be unequivocally hard Eurosceptical as had other western European populist radical right-wing parties with which it has been allied at the European level. The party never formally expressed a blanket and principled opposition to European integration. Rather, it called for reforms and changes that were not consistent and specific enough to establish a clear alternative vision of the EU, and indeed equivocated on whether the EU should remain in existence or not. In other words, the LN practiced an “equivocal Euroscepticism” allowing the party to present itself as deeply critical of the EU while ultimately accepting governmental responsibilities (Heinisch et al., 2021).

The painful fiscal consolidation efforts by the Monti government were also staunchly opposed by the Five Star Movement (FSM), a new populist actor that emerged in 2009 (Lanzone & Woods, 2015). It benefited from the permeability of both centre-right and centre-left coalitions to corruption, whether true or perceived, which fed the protest against “the establishment”. The FSM was set up as a full-fledged party in 2009 by the former comedian Beppe Grillo, who built the party on the success of his personal blog, and by the founder of a small IT company, the self-styled visionary Gianroberto Casaleggio. In his political initiative, Grillo successfully combined the rhetoric of direct democracy (“Uno vale Uno” or “Everyone is an Equal” in the FSM) with a firm grasp over the party and relentless imposition of party discipline. The FSM gradually developed a pronounced Eurosceptic profile. The abolition of the EU Fiscal Compact (a treaty set up in the aftermath of the Greek fiscal crisis which hamstrings the room for manoeuvre of EU Member States) was a key target of the party’s campaign. Whether to remain in the Eurozone or not, the party’s line was that the decision should be devolved to an unspecified ‘consultation’ among citizens (Font et al., 2021). The FSM entered parliament in massive numbers after the 2013 national elections but refused to enter any coalition with mainstream parties. In line with the mobilization of the most electorally successful ‘inclusionary’ populist contender parties in Southern Europe over the post-crisis period, the main narrative in Five Stars’ political discourse revolved around a financial oligarchy, seen to be in concord with dictates stemming from Germany, and trampling on the rights to a better life for European citizens. A strong critique was directed at the EU, questioning its very legitimacy and calling for Italians to take back their sovereignty (Caiani, 2019). Yet, the FSM did not turn into a hard Eurosceptic party. Rather, the party leadership made efforts to de-emphasize its Euroscepticism by issuing statements that denied it would be non-compliant with EU rules in the event FSM won the 2018 elections and entered government as the majority party.

The latest Italian party to join the populist family is the radical-right Fratelli d’Italia (Brothers of Italy) which spun off from the Berlusconi-led People of Freedom party in 2012. It draws on the legacy, resources and rank-and-file of the neo-fascist Italian Social Movement (established in the aftermath of WWII) and its successor National Alliance which was founded in 1994 attempting to distance itself from its post-fascist legacy). This “new” party combines a traditional national-conservative agenda with an anti-immigration and Eurosceptic stance (see Taggart & Pirro, 2021, who classify this party as ‘populist’—see also the datasets by Rooduijn et al., and Bakker et al., accessed 2022).

The party system in Italy is thus composed of quite a number of populist parties. Their combined electoral share at the general elections held in 2018 was 54.4% of the electorate if we exclude the electoral share of Berlusconi’s FI, and 68.4% if the votes of FI are added. The corresponding composition in terms of parliamentary representation is even slightly higher due to a majoritarian bias in the electoral law. This populist underpinning of the Italian political system was now the reality with which the EU’s new economic governance which took shape in 2020 would be interacting.

## A major shift in the economic governance of the EU

It is possible to identify three major phases in the changing of the EU economic governance over the pre- and post-pandemic period starting with the establishment of the new College of Commissioners of the European Commission and commencing with the new legislative period after the May 2019 elections of the European Parliament. The first phase comes after the new Commission took office in late 2019, upon receiving the vote of confidence from the European Parliament, and emphasizes changes in policy priorities for the incoming government at the EU level. These priorities markedly emphasised the green and digital transitions, and corresponding reforms of these priorities at the Member State level. While by themselves these policy priorities were not especially new, the goals were set to be quite ambitious. Notably, these priorities were maintained in full even after the onset of the pandemic. It is important also to note that the European “Green Deal” plan was to include a sustainable investment strategy that would necessitate a significant degree of flexibility in public spending partly overshadowing earlier EU fiscal constraints imposed by the SGP.

In early February 2020, the European Commission presented a review of the effectiveness of the SGP. A debate on the future of the economic surveillance framework was launched right when the pandemic struck Europe the hardest, in February–March 2020, and regulation on state aid was waived. To better appreciate this change, it is worth considering the centrality of antitrust and state aid rules, which are at the core of the functioning of the European single market (Di Mascio et al., 2020; Mascio et al., 2020). A second important step was a relaxation of budget rules, announced on 23 March 2020 after EU Finance Ministers agreed to use the “general escape clause” which can be triggered when “a severe economic downturn in the euro area or the Union as a whole” occurs, to suspend the obligations of the SGP and enable Member States to take “all necessary measures” to protect the health of the people and the economy, with the caveat that such measures were to be temporary and targeted.

The third phase, and the biggest governance shift, occurred over April-July 2020, when the new EU economic governance took shape, allowing the EU for the first time to collectively borrow on the financial markets on a fairly massive scale to support the economic recovery of the EU as a whole, targeting the larger amounts of investments towards the countries most afflicted by the pandemic. Crucially, debt was to be reimbursed through EU-wide taxes and tariffs. Jointly, these changes amounted to a potentially transformative shift in EU economic governance.

This major shift in EU economic governance can be explained through a quest for consistency with already developed theorising in the field of public policy and administration (Dunlop et al., 2020). The framework of this change consists of three core components: (i) the opening of a window of opportunity for policy change; (ii) political leadership, notably from then Chancellor Merkel of Germany, making the most of the opportunity for pushing through change; and (iii) inter-organisational and policy learning accumulated over the previous years and accelerated by the crisis mode which enabled the formulation of the new policy. Applying the framework to the facts of this case is discussed below.

First, an opportunity window was suddenly and dramatically opened by the outburst of the COVID-19 epidemic. The opportunity arising from the pandemic was seized by political leaders to shape the EU response and effect a major policy change. These dynamics can be analysed according to the model of policy change articulated by John Kingdon whereby ‘opportunity windows’ for radical policy change get opened up at the confluence of political, policy, and problem streams (Kingdon, 2004). Notably, the rifts that arose among EU Member States at the outburst of the Covid-19 epidemic triggered a ‘crisis’ with serious portents for the EU. The policy issue (Kingdon, 2004) came to be framed in terms of an emergency occurring on top of other sets of interconnected emergencies that had unfolded during the previous decade (Wolff & Ladi, 2020) leading thereby to a deeper crisis that threatened the EU which was already under stress from the aftermath of the deep financial induced recession of 2008, the inflow of migrants, and to some extent Brexit.

The issue of how to respond collectively at the EU level thus became salient to the policy agenda of EU decision-making institutions (political stream). The problem came to be framed in terms of how to ensure that the asymmetric form in which the pandemic struck the individual Member States of the EU would not lead to the very breakdown of the institutional architecture of the EU (problem stream). The policy option which came to be prioritised was to channel resources to those EU Member States which were most vulnerable to another economic adjustment after the previous austerity period, with per capita grants allocated corresponding to past economic vulnerabilities of the recipient countries (policy stream), as thoroughly demonstrated by Armingeon et al. (forthcoming). As noted, German Chancellor Merkel, President of the EU Commission Ursula von der Leyen, and French President Emmanuel Macron were important actors in ameliorating stringent public finance conditions previously imposed throughout the Eurozone (Ferrera et al., 2021). The second key component, therefore, in ameliorating these conditions in order to preserve the cohesion of the EU political-institutional system was political leadership (Hartley, 2012). Political leaders consolidated the foundations of the EU through exploiting the situational challenges arising from the financial and other consequences of the COVID-19 pandemic.

A third explanatory component was the process of learning that had been building up prior to the COVID-19 pandemic. The learning process occurred both within and across key organisations of the EU apparatus. The review of economic governance by the EU Commission found that EU formal procedures guided Member States to achieve fiscal targets. Despite this, public debt remained high in Member States such as Italy that had failed to experience significant economic growth. In these countries, mostly in southern Europe, choices were made internally to increase current expenditures rather than to protect investment to comply with EU formal procedures. Reform momentum had faded in these countries, such as Italy, because austerity policies prompted the rise of populist parties. Notably, the new Directorate for Reform of the European Commission provided an institutional resource in the EU polity embodying the new EU approach to public sector reforms: afield from the previously all-encompassing cutback logic and orientated towards a facilitating, supporting role performed by EU institutions. Drawing on the experience in building capacity in Member States, the Technical Support Instrument (TSI) was launched as a continuation of the SRSS. EU Member State can request tailor-made advice under the TSI not only to implement reforms in the context of EU economic governance, such as those arising from country-specific recommendations under the European Semester but also to prepare, amend, implement and revise national recovery and resilience plans under the Recovery and Resilience Facility (European Commission, 2020). These intra- and inter-organisational learning processes performed an enabling role facilitating decisions entailing a radical departure from the previous situation to occur. Thus, intra- and inter-organisational learning is a complementary explanatory factor, in the perspective of organisational learning theories and their significance for public policy (Dunlop & Radaelli, 2018).

To sum up, the public and policy debate that stemmed from the 2008 financial, economic and fiscal crises in Europe was shifting away from the logic of controlling public deficit and debt at any cost (literally), the so-called ‘austerity’ policy, and towards a recognition of the necessity for deficit-spending in order to sustain the economy and to counter the social and economic grievances that were driving a growing public sentiment for the anti-system, populist parties. In other words, a logic of conditionality in intra-EU fiscal relations was, slowly but assuredly, being replaced by a logic of solidarity as a result of learning from the failures of fiscal austerity. We now turn to consider the impact of the changed EU economic governance—that is, altered EU adjustment pressures—on national-level public sector reforms, zooming in on the case of Italy.

## The influence of the shift in EU economic governance on public sector reforms: the case of Italy

The Italian general elections held in March 2018 marked a turning point in the transformation of the Italian party system. They resulted in a hung parliament ultimately leading to the formation of a full-fledged populist cabinet, backed in Parliament by the Five Star Movement and the Northern League. This outcome followed three months of posturing, drama and negotiations going on behind closed doors and fits a broader pattern of political instability in Italy (Mele & Ongaro, 2014), especially as a consequence of the Eurozone crisis. The centre-right coalition, whose main party was the League, held the most seats but was far from a majority. The FSM became the party with the largest number of votes, while the centre-left coalition, that had the parliamentary majority for the incumbent government, came in third. EU fiscal constraints had operated for the Gentiloni government, which maintained a pro-integrationist approach and took forward structural reform process in line with EU guidelines. Conversely, EU constraints were rejected by the FSM and LN, which expressed radical criticism of the Eurozone governance. Both parties conducted a relatively Eurosceptic campaign preceding the 2018 elections even as their leaders often-times stressed their aim to reform the EU from within rather than prioritizing a ‘Euroexit’ policy, or at least conveyed mixed messages in this regard.

In the aftermath of the 2018 elections, the FSM refused to negotiate over a new government with FI because of the numerous judicial scandals involving its leader Silvio Berlusconi. This broke the centre-right coalition and finally led to a deal between the FSM and the League, giving rise to the first governing coalition of exclusively left- and right-wing populist parties to the extent the left–right spectrum holds explanatory power to characterize populism. The new government consequently had a peculiar configuration as a “three-headed” cabinet: the leaders of the FSM and the League—Luigi Di Maio and Matteo Salvini respectively—were appointed as deputy Prime Ministers, while the non-partisan outsider Giuseppe Conte, a university professor and active lawyer, was appointed as Prime Minister, whose primary task was ensuring coordination between the coalition partners. Tensions arose between governmental leaders and the President of the Republic, Sergio Mattarella, concerning the appointment of certain members of the Cabinet. In particular, the proposed Minister of the Economy and Finance, Paolo Savona, was known for his support for Italy’s exit from the Euro. Eventually, Savona was appointed as Minister for European Affairs, while the key role of Minister of Economy and Finance was entrusted to Giovanni Tria, a technocrat with no anti-euro reputation. The FSM and the League tried to reconcile policy divergences by means of a formal coalition agreement known as the “contract for the government of change”. In this document, the government committed itself to promoting public investment, maintaining that the European Commission should not take these investment costs into account when calculating the deficit. Moreover, the agreement expressed a radical critique of the Eurozone governance and committed the government to demand reform of the EU treaties.

Yet, the agreement did not prevent programmatic divergences between the coalition partners to become publicly known. Notably, the FSM pledge to implement a universal scheme of basic income was hard to reconcile with the League’s aim to introduce tax cuts and scrap the pension reform that had raised the retirement age under the Monti government. These reform proposals were very expensive and implied tough budgetary choices that were in open conflict with EU rules. The high level of public debt implied by these postures if enacted would challenge Italy’s ability to maintain easy access to borrowing on the financial markets. Moreover, the same consequences for low cost borrowing would adversely affect deficit-spending of any government in the same fiscal position as that of Italy. It became apparent in the winter of 2018 that the fiscal space for the reform proposals of the two parties was not available unless the government decided to confront Brussels head on and by so doing risk a storm on the financial markets. Italy’s domestic vulnerability to heightened tensions on the financial markets was a consideration in the government’s decision to adopt a more prudent fiscal position. In this regard, threats from other countries in the Eurozone in support of the European Commission’s determination to take legal action for deviation from the SGP’s deficit rules induced the Conte government to adopt a more prudent fiscal position (Fabbrini & Zgaga, 2019).

When it became clear that the Conte government was willing to step back from its hard-line stance and revise the budget to comply with the Eurozone rules on fiscal discipline, a new phase of the Conte government began. The EU could be blamed and used as a scapegoat by the League, which became Italy’s largest party in the 2019 European Parliament elections in which the FSM lost more than one third of its electoral support. The electoral results intensified internal divisions within the Conte cabinet, which became fully evident in the formation of the new European Commission and in particular in the selection of the new Commission president in the summer of 2019. While the selection of the President of the European Commission is made by the heads of member governments of the EU, the selection requires confirmation by the European Parliament. The members of the European Parliament of the FSM voted in favour of the mainstream candidate Ursula von der Leyen for the position of Commission president, also in order to allay their political isolation within the European Parliament, whereas the League’s MEPs voted against her (Cotta, 2020). Ms Von der Leyen was eventually confirmed by the Parliament, with the decisive votes of the FSM. Environmental policy became an important fault line between the FSM and the League as the FSM moved farther away from the League on this issue. The two parties took opposite positions on the “European Green Deal” which was a landmark legislative package of the new Commission. While environmental sustainability was a core value of the FSM since its inception, environmentalism was barely featured in the domestic 2018 election campaign. The effort by the FSM to reposition itself on the new European political landscape helped put the environment back on the domestic agenda.

The League tried to turn internal divisions within the Conte cabinet to its favour with a demand for early elections. The Italian parliamentary system allows for different coalitional majorities to be formed in Parliament. A new majority was thus constituted in the summer of 2019, by associating the FSM with the main opposition party, the centre-left and pro-EU Democratic Party. Conte was confirmed as Prime Minister of this new government. The Conte II government repositioned Italy in the mainstream of EMU Member States. This was signified by its proposal to appoint Paolo Gentiloni, the Prime Minister of the pro-EU centre-left government preceding the 2018 Italian elections, as a European Commissioner. Also, a prominent pro-EU Italian MEP, Roberto Gualtieri, previous chairman of the influential European Parliament’s Economic and Monetary Committee, was appointed as Minister of Economy and Finance. In a few months, budgetary policy was normalized and became fully compliant with EMU rules for fiscal discipline. As a result, the pressure from financial markets also faded away (Fabbrini, 2021).

Still, in this initial phase there was little public support for the most recent Conte government, reflecting divisions between the coalition partners. These divisions were further heightened by the formation of a splinter party, Italia Viva (IV) roughly translatable as “Italy is alive and lively”. This fringe centrist part was established by the former leader of the Democratic Party, Matteo Renzi, who left that party in autumn 2019. A fear that the Conte II government would collapse due to IV’s holding a decisive number of senators that could vote the government out of office, faded with the outburst of the COVID-19 pandemic in March 2020. The public health crisis led to a major shift in the EU’s economic governance as we observed earlier. The so-called general escape clause of the Stability and Growth Pact (SGP), regulating public expenditures and state aid, was triggered.

This did not suspend the procedures of the SGP but allowed the Conte II government to depart from the fiscal requirements that would have normally applied. The government adopted expansive counter-cyclical budgetary and other measures, to sustain the healthcare system and provide relief to those citizens and businesses that had been particularly impacted. Dozens of “fast-track”, temporary procedures were bundled together in several rounds of emergency packages, with benefits distributed to a plethora of recipients. This approach reproduced the shortcomings of the historical fragmentation of economic assistance in Italy in the form of special provisions that target particularized interests while simultaneously making it difficult to steer and oversee the implementation of such multiple procedures (Di Mascio et al., 2020; Mascio et al., 2020).

The governing coalition was too fragile and conflict-ridden to implement any package of structural reforms. With growing public support owing to the successful negotiations at the European level concerning the Resilience and Recovery Fund making available to Italy an unprecedented amount of EU-backed 209 billion euros over a 6-year period, the Prime Minister convened a public meeting to gather ideas from interested parties about how to use the EU funds. The PD and IV criticized the Conte government for not tapping the EU’s bailout fund—the European Stability Mechanism (ESM)—to shore up its healthcare service. This issue reignited the conflict of these coalition partners with the FSM, which resisted the ESM as anathema to its Eurosceptic roots. The FSM pushed a referendum held in September 2020 which led to the reduction of the number of MPs in both chambers of the Italian Parliament. The victory of the “yes” vote in this referendum subsequently intensified the rivalry between the coalition partners, amidst growing social unrest resulting from the restrictive measures designed to contain the second wave of Covid-19 infections in the fall of 2020.

Eventually, the Conte II government collapsed in January 2021 when the IV opposed the draft National Recovery and Resilience Plan (NRRP) advocated by the Prime Minister. Renzi’s party lamented the lack of a longer-term perspective on economic reconstruction amidst growing uncertainty over Italy’s future. It pulled its support over plans for a task force to oversee the implementation of the plan, claiming that the creation of a parallel structure would bypass representative institutions. After the resignation of Conte as Prime Minister, the President of the Republic issued an appeal to all parties represented in Parliament to support the formation of a government led by Mario Draghi, a high-profile technocrat who held a PhD from MIT in the US and was a former president of both the Bank of Italy and the European Central Bank, amongst other public roles. The Draghi government won the support of all the parties in Parliament except Brothers of Italy, and a fringe faction of the FSM. Although Draghi put emphasis on greater European integration, his cabinet was backed also by the League, whose leader argued that a figure with Draghi’s imposing authoritativeness would give Italy more sway in EU policy-making. The League also needed to meet the wishes of business leaders in the party’s power bases in Northern Italy, who urged the party to support the new government and help distribute the EU funds.

The new government displayed a majority of partisan ministers, but the key ministries were assigned to technical experts. The legitimacy of these technocrats was also provided by the EU and its Member States, which supported the government turnover via the channel of backroom diplomacy. This was not an unprecedented twist of the TLG, since EU-level actors had already influenced the appointment of the technocratic government headed by Monti in late 2011 (De La Porte & Natali, 2014). Unlike what happened in 2011, backroom diplomacy influenced the formation of a technocratic government, in a situation where EU adjustment pressures did not comprise formal procedures, since the SGP was suspended. Furthermore, the disbursement of recovery funds has not been tied to strict conditionality mechanisms: less stringent adjustment pressure made the rapprochement between the League and governing parties easier in combination with the rise in the polls of the populist radical right party (Brothers of Italy), which called for new elections immediately.

In late April 2021 the Draghi government submitted to the European Commission the revised version of the NRPP. The new version maintained the focus on the digital and green transition but also included some novelties. First, Italy planned to spend not only EU funds but also national ones (for a total amount of 236 billion euros) to revive its COVID-19-battered economy. Second, each intervention was accompanied by detailed operational programmes, with a view to ensure fine-grained monitoring by the Commission. Third, it included an ambitious reform programme focused on four areas: public administration, the justice system, simplification of legislation and competition.

## Discussion and conclusion

Our analysis has shown that in the context of the COVID-19 crisis, less stringent EU adjustment pressure was the result of an iterative process, where EU and domestic actors interact at the EU- and national-level game boards. Our empirical evidence has also highlighted that the institutional configuration of the EU, which makes the EU an actor (a set of actors) in its own right, and not just a forum or arena where national-level actors interact, has been crucial in shaping EU-domestic interactions in the process of public sector reform under conditions of the rise of populist parties and the responses to it by mainstream political actors.

These dynamics have led to a new economic governance in the EU. The seizing by EU leaders of the opportunity, in the context of the COVID-19 crisis, to change the EU economic governance has been catalysed by the process of learning that had been triggered by the fiscal crisis. In those countries like Italy that were deeply affected by austerity policies, citizens perceived a growing loss of autonomy of national governments vis-a-vis the EMU institutions, and they opted for populist parties, which voiced an opposition to the external constraints imposed by EU membership on the national economic policy. As a result, the rise of populism made EU policy makers aware that austerity policies had diminished the acceptance in large segments of the public opinion of the pre-2020 economic and fiscal surveillance framework, and this had occurred well before the outbreak of COVID-19. Learning processes about EU governance, that have occurred since the EU economic and fiscal crisis of 2009–14, have largely shaped the response by all actors, and most notably by pro-EU, “European establishment” actors, to the new crisis determined by the COVID-19 pandemic.

Within the frame of the new EU economic governance, EU-level actors and pro-EU domestic parties have been able to coalesce around a package of reforms, which are part and parcel of the National Recovery and Resilience Plan to implement at the domestic level the EU Next Generation, that seem to reflect and incorporate some stock-taking from previous reform exercises. Organisational and policy learning (Dunlop & Radaelli, 2018) from the previous crisis, that is, the economic and fiscal crisis of 2009–2014, is a key feature that shaped the response to the pandemic crisis. This finding, detected by this paper specifically in the case of Italy and in the Two-Level Game at the EU-Italy interface, may represent a more general finding pointing to the interconnectedness of successive crises over time—even when they occur in policy domains distinct, as the economic-fiscal policy on one hand and the health policy on the other—in shaping the EU governance, with learning providing the key linkage between successive crises.

However, the shift to a new economic governance in the EU has not implied a major restructuring of policy-making patterns in Italy. The malleability of populist parties on the issue of European integration has been greatly enhanced by the mutating character of Italian populism (Di Mascio et al., 2021), in a context marked by the fragmentation of the party system. Italian populist parties, depending on the circumstances and perceived electoral convenience, compete with each other in an unrestrained manner to position as representative, even standard bearer, of the various interests at stake, and in some instances they had openly allied or conflicted with each other without qualms about the coherence with positions previously supported. Basically, populists push for structural reforms only in an instrumental way, in order to gather consensus among economic and social categories, rather than because they are driven by a consistent ideology and vision of the state. On the recipes to be proposed for the solution of collective problems they are extremely fluid, supporting the tactical conveniences of the specific political phase. Traditional parties that failed to erode the consensus of populist parties ended up following their political agenda, emulating their communication methods and participating in a game of alliances with one or another populist force. With respect to public sector reforms, this led to considerable inconsistency in governmental action.

In the episode of major change narrated in this paper—the shift in EU economic governance and its consequences for the dynamics of public sector reforms—learning has led to pro-EU political actors to act strategically in ways very different from the past (Armingeon et al., forthcoming), but it may be conjectured that the dynamics of the Two-Level Game at the EU-Member States interface will be shaped by learning-driven countermoves by populist actors in the future. Since Italian (and other countries’) populist parties are not necessarily nationalist, but neither are they pro-European, they adopt a shifting, if not outright wavering, relationship with Europe that adapts by reacting to the strategies pursued by European-level policy-makers and that is driven by domestic electoral convenience. Italian populist parties may swerve from frontal opposition to Brussels to intense bargaining and then to moments (usually short-lived) of full cooperation. However, in the event that opportunities arise for the exploitation of anti-EU sentiments for purposes of political-electoral consensus, it appears quite plausible that Italian populists would return to nationalist positions, albeit this will possibly lead to populist actors playing their game differently than what they did during the 2010s. Learning processes—at individual, organisational, policy and political level—will be key in shaping the dynamics of the Two-Level Game that will be played in the 2020s and beyond in the EU.
